# Supplementary material for: Co-inhibition of ATM and ROCK synergistically improves cell proliferation in replicative senescence by activating FOXM1 and E2F1
Source: Commun Biol. 2022 Jul 14;5:702. doi: 10.1038/s42003-022-03658-5 (PMC9283421; doi:10.1038/s42003-022-03658-5)

Supplementary Information

Co-inhibition of ATM and ROCK synergistically improves cell proliferation in replicative senescence by activating FOXM1 and E2F1

Eun Jae Yang[1†], Ji Hwan Park[1†], Hyun-Ji Cho[1], Jeong-A Hwang[1], Seung-Hwa Woo[1], Chi Hyun Park[2], Sung Young Kim[3], Joon Tae Park[4], Sang Chul Park[5,6,*], Daehee Hwang[7,*], and Young-Sam Lee[1,5,8*]

[1]Department of New Biology, DGIST, Daegu 42988, Republic of Korea

[2]Department of Computer Science and Engineering, Kangwon National University, Chuncheon 24341, Republic of Korea

[3]Department of Biochemistry, Konkuk University School of Medicine, Seoul, Korea.

[4]Division of Life Sciences, College of Life Sciences and Bioengineering, Incheon National University, Incheon 22012, Republic of Korea

[5]Well Aging Research Center, Division of Biotechnology, DGIST, Daegu 42988, Republic of Korea

[6]The Future Life & Society Research Center, Advanced Institute of Aging Science, Chonnam National University, Gwangju 61469, Republic of Korea

[7]Department of Biological Sciences, Seoul National University, Seoul 08826, Republic of Korea

[8]New Biology Research Center, DGIST, Daegu 42988, Republic of Korea

[†]These authors contributed equally to this work.

*To whom correspondence should be addressed:

E-mail: lee.youngsam@dgist.ac.kr (Y.-S. Lee), daehee@snu.ac.kr (D. Hwang), or scpark@snu.ac.kr (S.C. Park).

Running Title: synergistic senomorphism by the combination of KU-60019 and Y-27632

Key Words: cellular senescence, senomorphism, cell cycle, FOXM1, E2F1

This file includes:

Supplementary Figures 1-14

Uncropped original western blots

Supplementary Figures

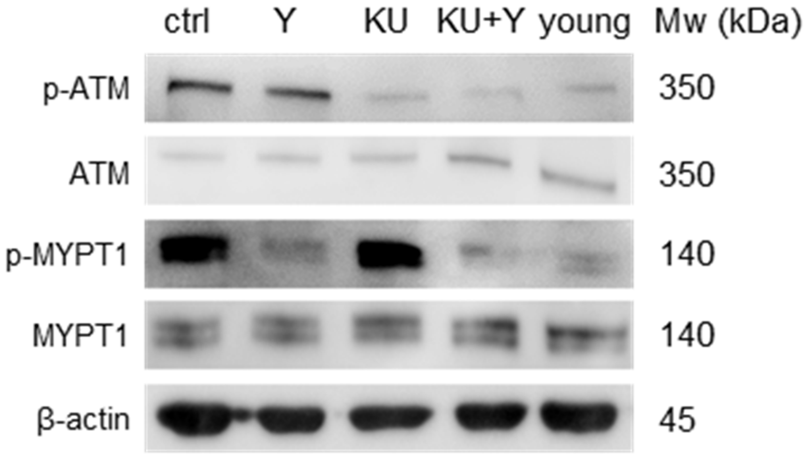

Supplementary Figure 1. Kinase inhibitions of ataxia-telangiectasia mutated (ATM) and Rho-associated protein kinase (ROCK) by KU and Y. Immunoblots showing the levels of phosphorylated ATM and MYPT1 at 1 hour after treatment with KU, Y, or KU+Y.

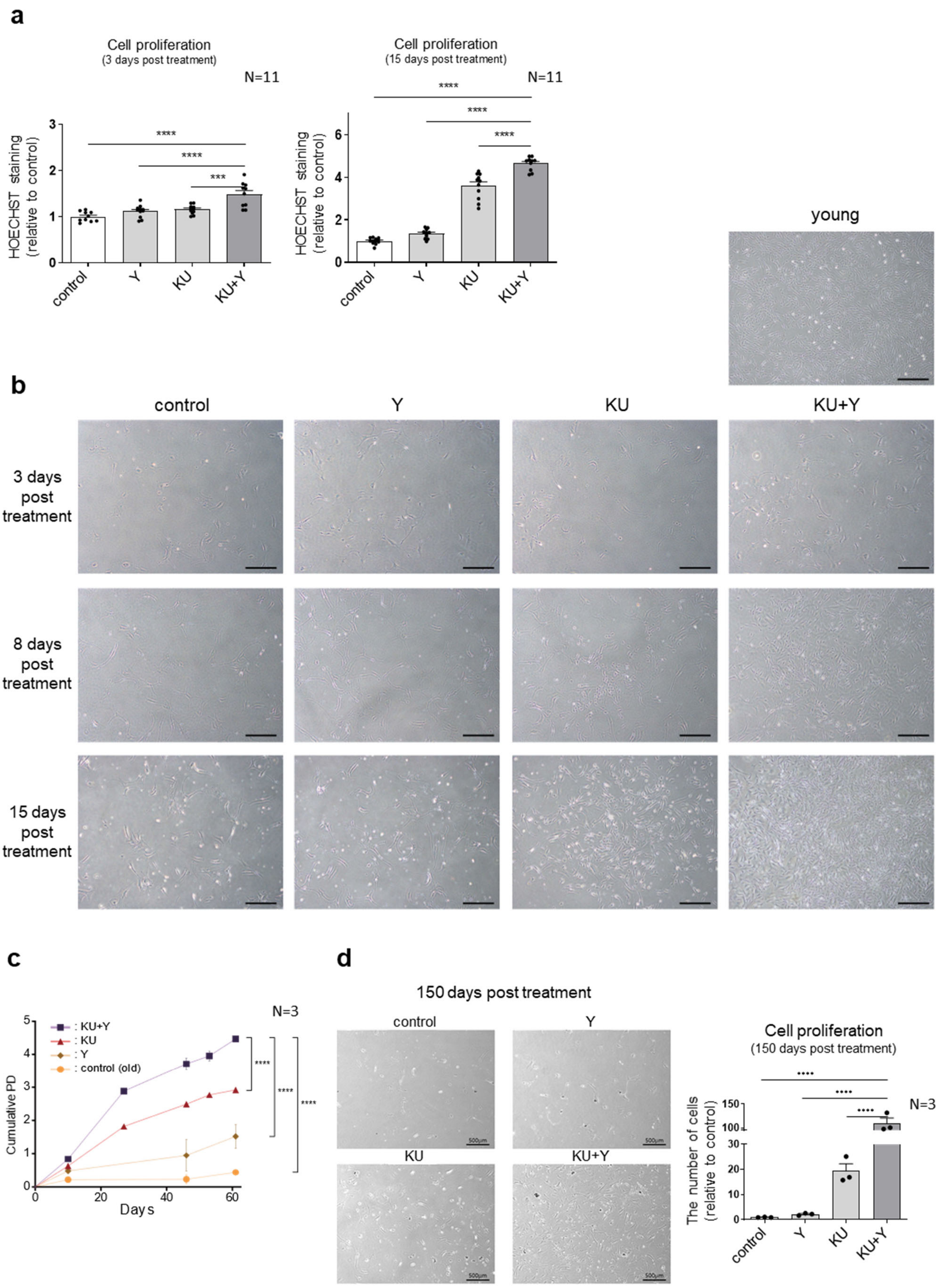
a
Cell proliferation
(3 days post treatment)
N=11
HOECHST staining (relative to control)
Cell proliferation
(15 days post treatment)
N=11
control
Y
KU
KU+Y
young
b
control
Y
KU
KU+Y
3 days post treatment
8 days post treatment
15 days post treatment
c
N=3
: KU+Y
: KU
: Y
: control (old)
Cumulative PD
Days
d
150 days post treatment
control
Y
KU
KU+Y
Cell proliferation
(150 days post treatment)
N=3
The number of cells (relative to control)

Supplementary Figure 2. Synergistic induction of cell proliferation by KU+Y. (**a**) Effects of KU, Y, and KU+Y on cell proliferation at 3 and 15 days post treatment (DPT). The data are shown as the mean ± s.d. values; N = 11 per experiment. ***, $P < 1.0\times10^{-3}$; ****, $P < 1.0\times10^{-4}$ from one-way analysis of variance (ANOVA) with Tukey's post hoc correction. (**b**) Images showing changes in cell morphology and population at 3, 8, and 15 DPT of Y, KU and KU+Y. Scale bar = 500 μm (**c**) Cumulative population doubling (PD) over time after treatment with KU, Y, and KU+Y. The data are shown as the mean ± s.d. values; N = 3 per experiment. ****, $P < 1.0\times10^{-4}$ from one-way ANOVA with Tukey's post hoc correction. (**d**) Images showing changes in cell morphology and population at 150 DPT of Y, KU and KU+Y. Cell counts were quantified at 150 DPT (**d**, right). The data are shown as the mean ± s.d. values; N = 3 per experiment. ****, $P < 1.0\times10^{-4}$ from one-way ANOVA with Tukey's post hoc correction.

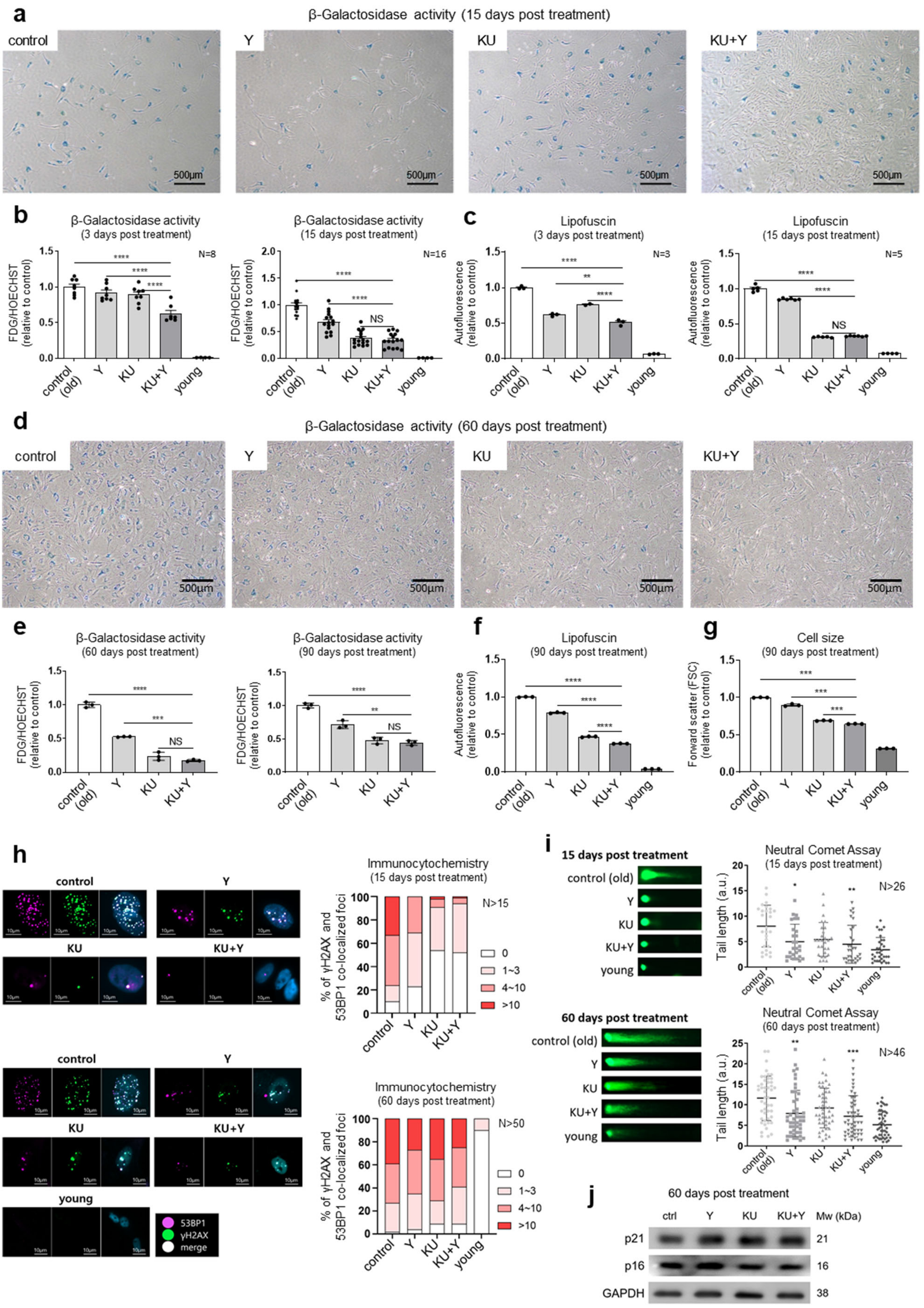
a
β-Galactosidase activity (15 days post treatment)
control
Y
KU
KU+Y
500μm
b
β-Galactosidase activity (3 days post treatment)
β-Galactosidase activity (15 days post treatment)
FDG/HOECHST (relative to control)
N=8
N=16
NS
c
Lipofuscin (3 days post treatment)
Lipofuscin (15 days post treatment)
Autofluorescence (relative to control)
N=3
N=5
control (old)
young
d
β-Galactosidase activity (60 days post treatment)
e
β-Galactosidase activity (60 days post treatment)
β-Galactosidase activity (90 days post treatment)
f
Lipofuscin (90 days post treatment)
g
Cell size (90 days post treatment)
Forward scatter (FSC) (relative to control)
h
Immunocytochemistry (15 days post treatment)
Immunocytochemistry (60 days post treatment)
% of γH2AX and 53BP1 co-localized foci
N>15
N>50
0
1~3
4~10
>10
53BP1
γH2AX
merge
10μm
i
15 days post treatment
60 days post treatment
Neutral Comet Assay (15 days post treatment)
Neutral Comet Assay (60 days post treatment)
Tail length (a.u.)
N>26
N>46
j
60 days post treatment
ctrl
Mw (kDa)
p21
p16
GAPDH
21
16
38

Supplementary Figure 3. Synergistic reduction of SA-β-galactosidase activity and lipofuscin amount by KU+Y. (**a**) Representative images showing SA-β-gal positive (blue) cells at 15 DPT. Scale bar = 500 μm (**b**) Effects of KU, Y, and KU+Y on FDG-based SA-β-gal activity of high passage HDFs at 3 and 15 DPT. The data are shown as the mean ± s.d. values; N = 8 or 16 independent experiments. (**c**) Effects of KU, Y, and KU+Y on lipofuscin-originated autofluorescence of high passage HDFs at 3 and 15 DPT. The data are shown as the mean ± s.d. values; N = 3 or 5 per experiment. (**d**) Representative images showing SA-β-gal positive (blue) cells at 60 DPT. Scale bar = 500 μm. (**e**) Effects of KU, Y, and KU+Y on FDG-based SA-β-gal activity of high passage HDFs at 60 and 90 DPT. The data are shown as the mean ± s.d. values; N = 3 independent experiments. (**f**) Effects of KU, Y, and KU+Y on lipofuscin-originated autofluorescence of high passage HDFs at 90 DPT. The data are shown as the mean ± s.d. values; N = 3 per experiment. (**g**) Effects of KU, Y, and KU+Y on forward scatter (FSC)-based relative cell size of high passage HDFs at 90 DPT. The data are shown as the mean ± s.d. values; N = 3 per experiment. For statistical analyses of data on **b-c**, and **e-g**, *, $P < 0.05$; **, $P < 1.0\times10^{-2}$; ***, $P < 1.0\times10^{-3}$; ****, $P < 1.0\times10^{-4}$ by one-way ANOVA with Tukey's post hoc test. (**h**) Representative images showing formation of γH2AX (purple) and 53BP1 (green) foci representing DNA damage in high passage HDFs at 15 and 60 DPT with KU, Y, and KU+Y. The percentage of colocalized H2AX and 53BP1 foci in a single nucleus was presented. (**i**) Representative images showing the length of comet tails representing DNA damage in high passage HDFs at 15 and 60 DPT with KU, Y, and KU+Y. The tail length was presented as the mean ± s.d. values. *, $P < 0.05$; **, $P < 1.0\times10^{-2}$; ***, $P < 1.0\times10^{-3}$ by one-way ANOVA with Tukey's post hoc test. (**j**) The protein expression levels of p21 and p16 at 60 DPT. GAPDH was shown as a loading control.

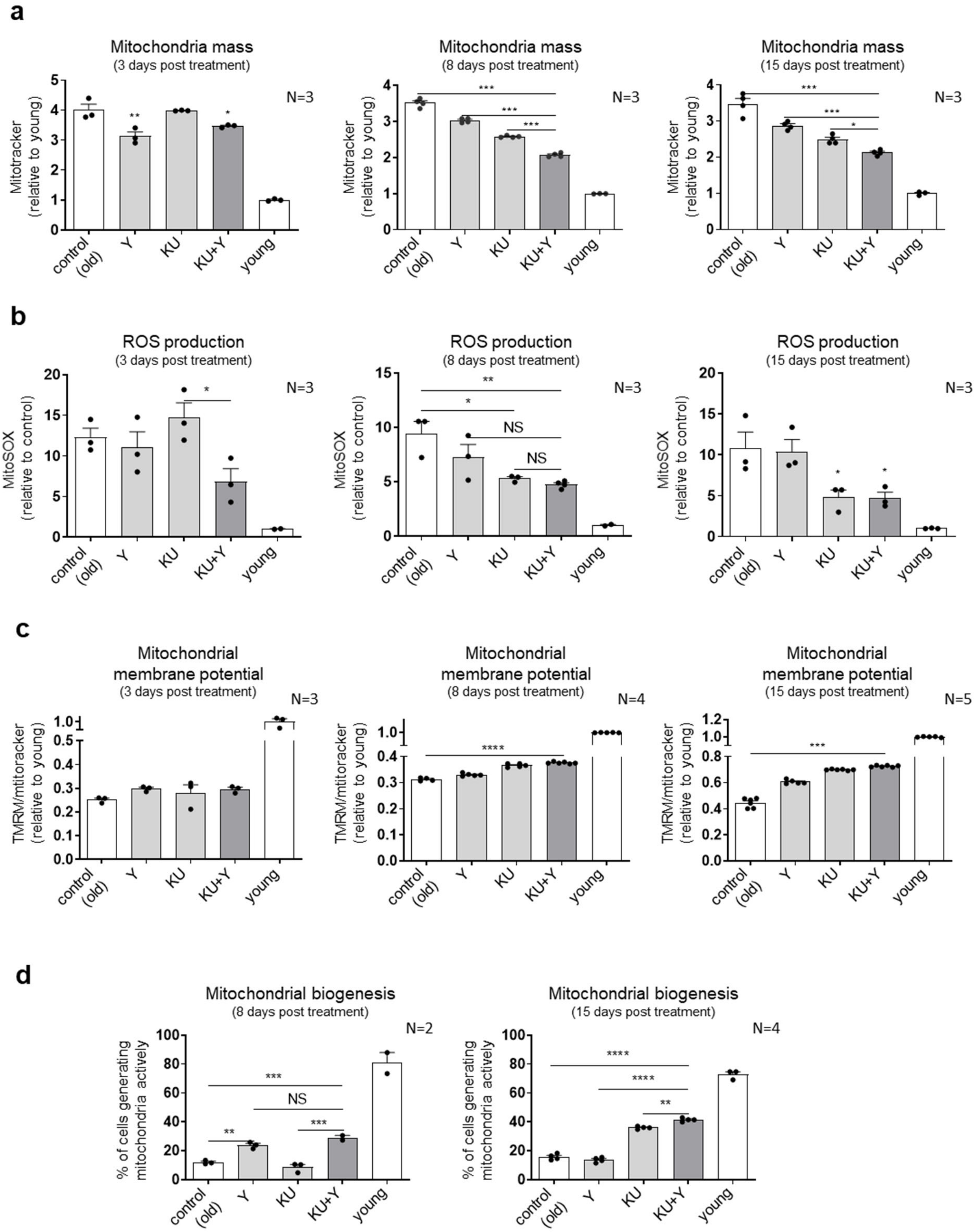

Supplementary Figure 4. Correction of mitochondrial impairment by KU+Y. (**a-d**) Effects of KU, Y, and KU+Y on mitochondrial mass (**a**), ROS production (**b**), and mitochondrial membrane potential (**c**) at 3, 8, and 15 DPT and on mitochondrial biogenesis (**d**) at 8 and 15

DPT. The data are shown as the mean ± s.d. values; N = 2-5 per experiment (see the corresponding plots). NS, not significant ($P > 0.05$); *, $P < 0.05$; **, $P < 0.01$; ***, $P < 1.0\times10^{-3}$; ****, $P < 1.0\times10^{-4}$ from one-way ANOVA with Tukey's post hoc correction.

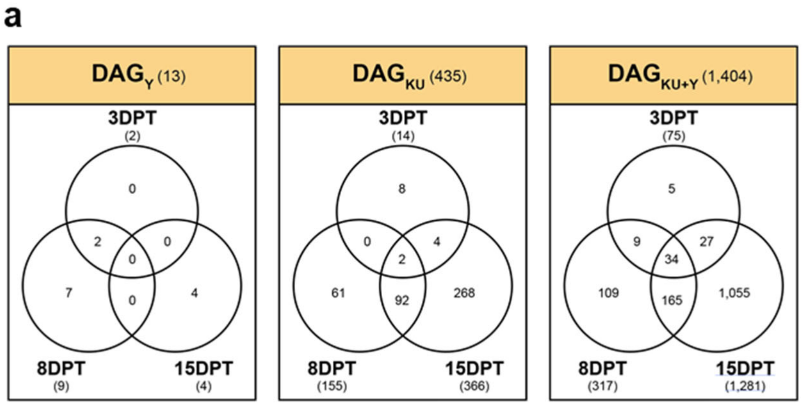

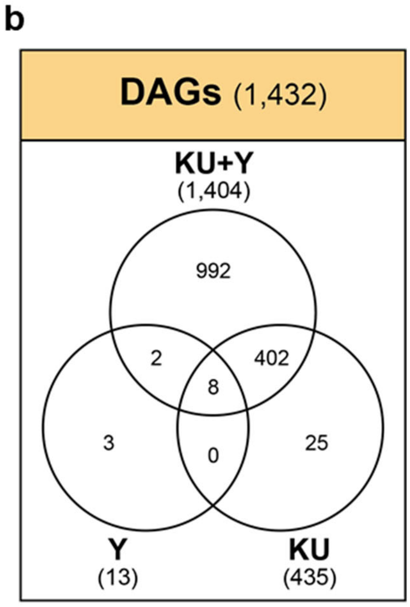

c

SAGs	DAGs$_{KU+Y}$	SynAG3	SynAG8	SynAG15	# of Genes	
up-reg.	down-reg.	down-reg.	-	-	**11**	→ E-DOWN
		-	down-reg.	-	**10**	→ M-DOWN
		-	-	down-reg.	**314**	→ L-DOWN
		down-reg.	down-reg.	-	3	
		down-reg.	-	down-reg.	3	
		-	down-reg.	down-reg.	1	
		down-reg.	down-reg.	down-reg.	2	
		-	-	-	293	
	-	-	-	-	1,795	
down-reg.	up-reg.	up-reg.	-	-	2	
		-	up-reg.	-	**105**	→ M-UP
		-	-	up-reg.	**377**	→ L-UP
		up-reg.	up-reg.	-	4	
		up-reg.	-	up-reg.	4	
		-	up-reg.	up-reg.	**68**	→ ML-UP
		up-reg.	up-reg.	up-reg.	**10**	→ EML-UP
		-	-	-	197	
	-	-	-	-	1,705	

Supplementary Figure 5. Identification of drug-associated genes (DAGs) and synergistic senomorphisim-associated genes (SynAGs).

(**a-b**) Venn diagram showing relationships among DAGs of each DPTs in KU, Y and KU+Y treatment (**a**) and drug treatment conditions (**b**). (**c**) Categorization of SynAGs based on up-(red) or down-regulation (blue) in the comparisons of KU+Y vs. DMSO-treated high passage HDFs (KU+Y/old); and KU+Y vs. KU (KU+Y/KU) or Y-treated (KU+Y/Y) high passage HDFs at 3 (SynAG03), 8 (SynAG08), and 15 (SynAG15) DPTs. The number of SynAGs are shown in the last column of the table.

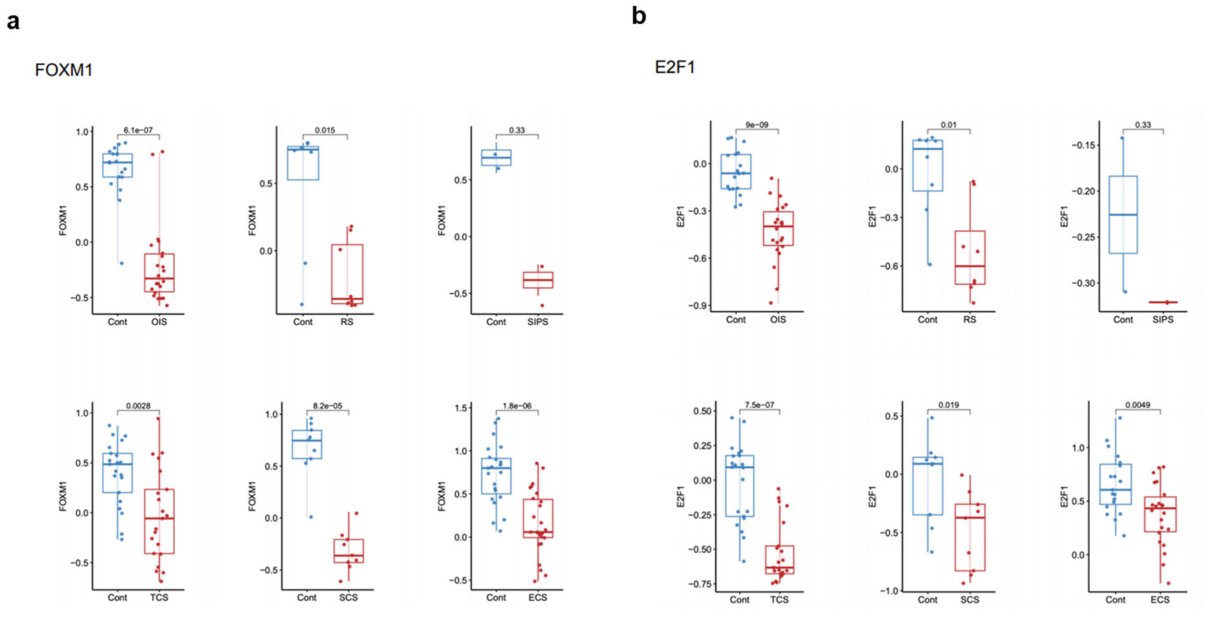

Supplementary Figure 6. Senescence-associated down-regulation of FOXM1 and E2F1 observed in various types of cellular senescence models. (**a-b**) Down-regulation of FOXM1 (**a**) and E2F1 (**b**) in oncogene-induced senescence (OIS), replicative senescence (RS), stress-induced premature senescence (SIPS), tumor cell senescence (TCS), stem cell senescence (SCS), and endothelial cell senescence (ECS). A *P*-value from Student t-test is indicated for each comparison.

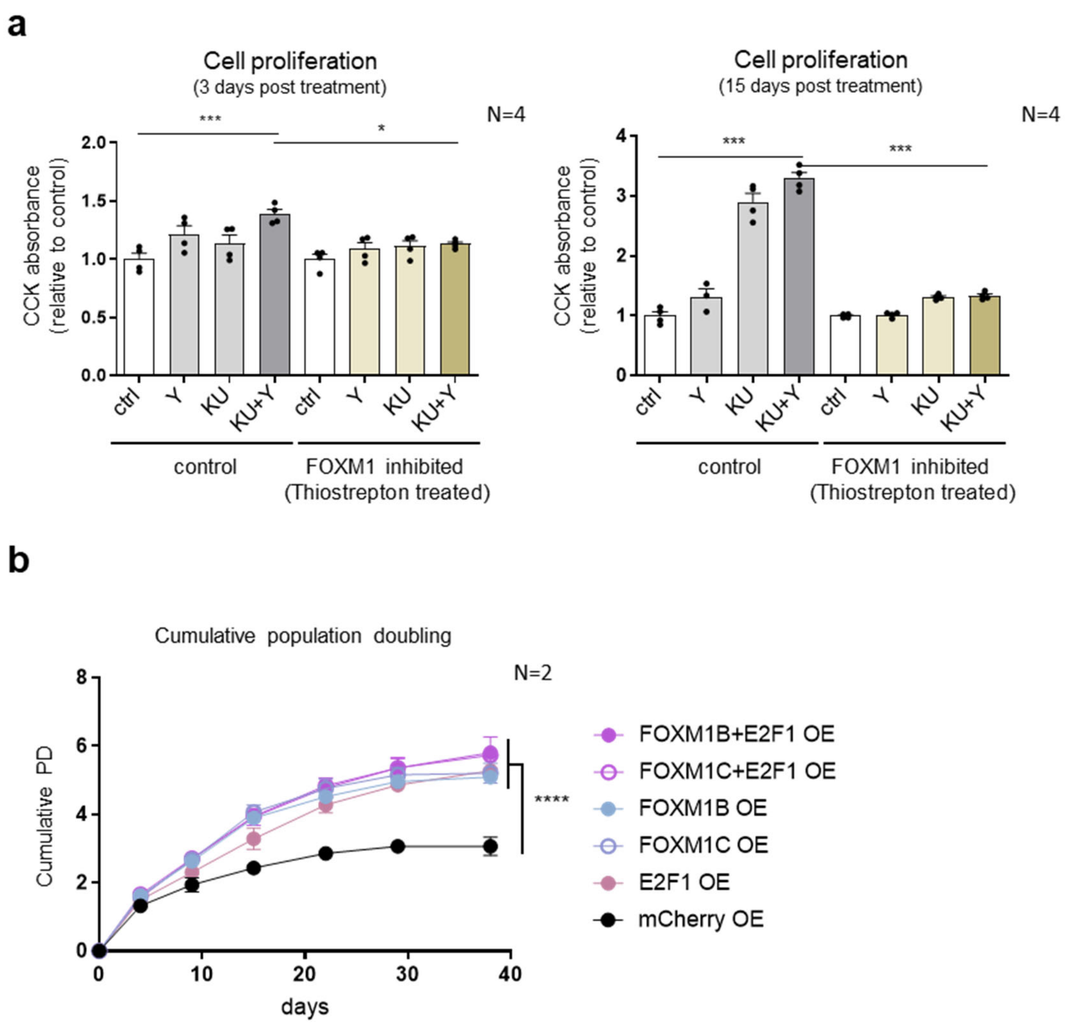

Supplementary Figure 7. Effects of FOXM1 inhibition and overexpression on cell proliferation. (**a**) Suppressed proliferation of KU, Y, or KU+Y-treated high passage HDFs by treatment of thiostrepton, an inhibitor of FOXM1. The data are shown as the mean ± s.d. values; N = 4 per experiment. (**b**) Cumulative population doubling (PD) of high passage HDFs overexpressing FOXM1B, FOXM1C, E2F1, FOXM1B and E2F1, FOXM1C and E2F1, or mCherry (control). The data are shown as the mean ± s.d. values; N = 2 per experiment. *, $P < 0.05$; ***, $P < 1.0\times10^{-3}$; ****, $P < 1.0\times10^{-4}$ from two-way ANOVA with Tukey's post hoc correction.

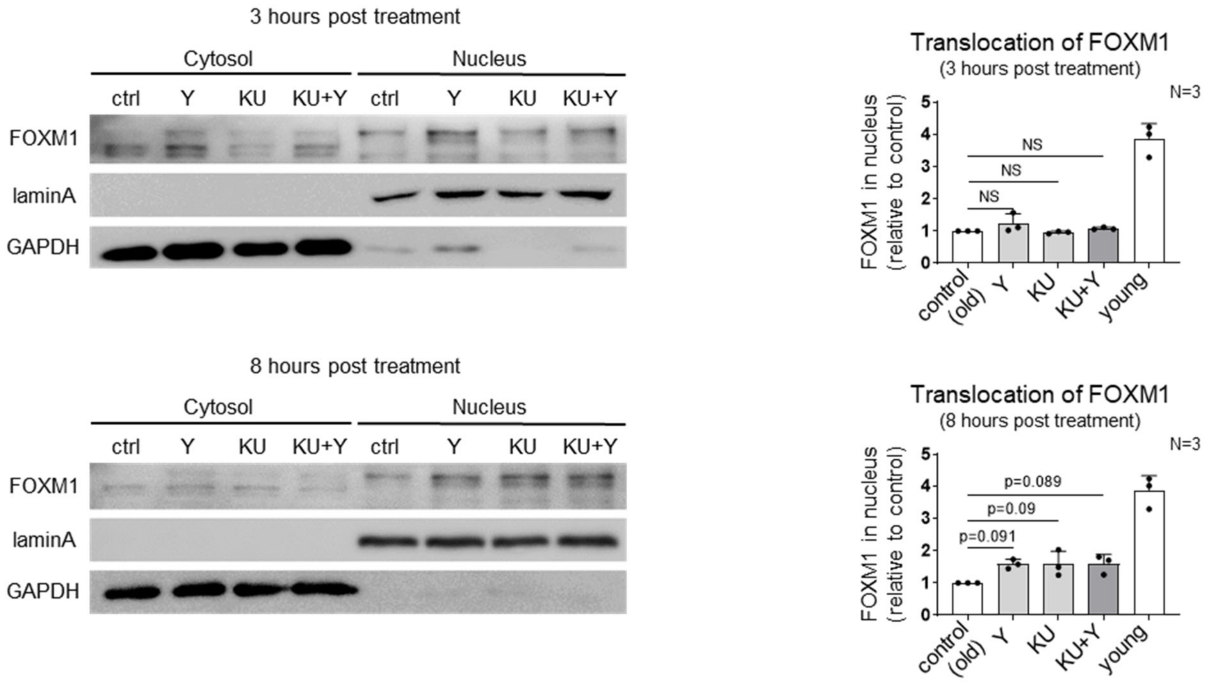

Supplementary Figure 8. Induced nuclear translocation of FOXM1 by KU+Y. Immunoblots against anti-FOXM1 antibody of cytosolic and nuclear fraction at 3 and 8 hours and 8 days after treatment with KU, Y, or KU+Y. Amounts of nuclear FOXM1 were quantified using the immunoblot images. The levels of lamin A and GAPDH were used to normalize the amounts of nuclear and cytoplasmic FOXM1, respectively. The data are shown as the mean ± s.d. values; N = 2-4 per experiment. *, $P < 0.05$ from one-way ANOVA with Tukey's post hoc correction.

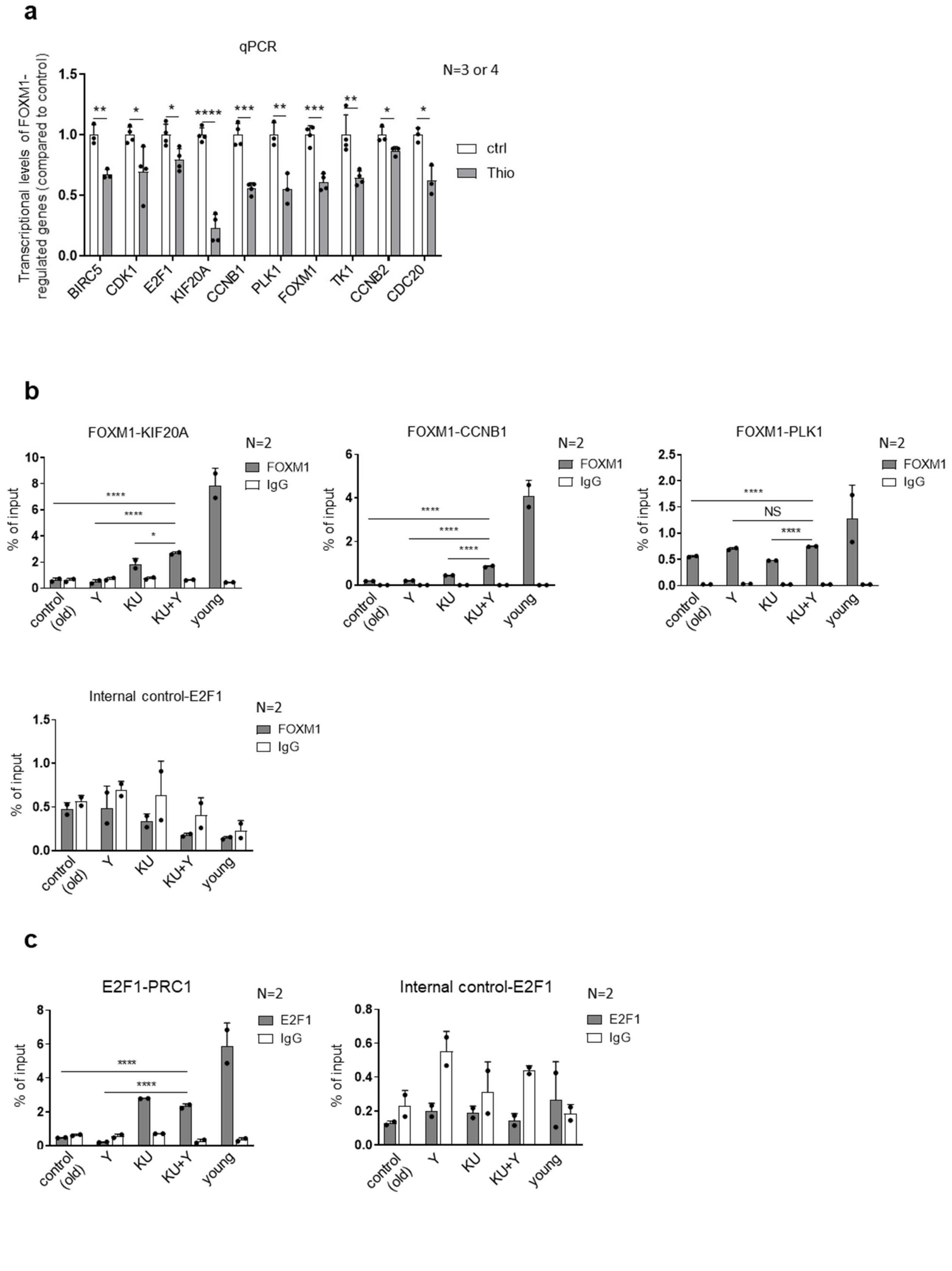

Supplementary Figure 9. Increased promoter binding of FOXM1 and E2F1 by KU+Y. (a) Transcriptional expression levels of FOXM1 targeted genes after treatment of thiostrepton. The

data are shown as the mean ± s.d. values; N = 3-4 per experiment. *, $P < 0.05$; **, $P < 1.0\times10^{-2}$; ***, $P < 1.0\times10^{-3}$; ****, $P < 1.0\times10^{-4}$ from two-tailed unpaired t test. (**b-c**) Binding strength of FOXM1 (**b**) and E2F1 (**c**) to the promoters of the indicated target genes measured by ChIP-qPCR analysis. The data are shown as the mean ± s.d. values; N = 2 per experiment. *, $P < 0.05$; ****, $P < 1.0\times10^{-4}$ by two-way ANOVA with Tukey's post hoc test.

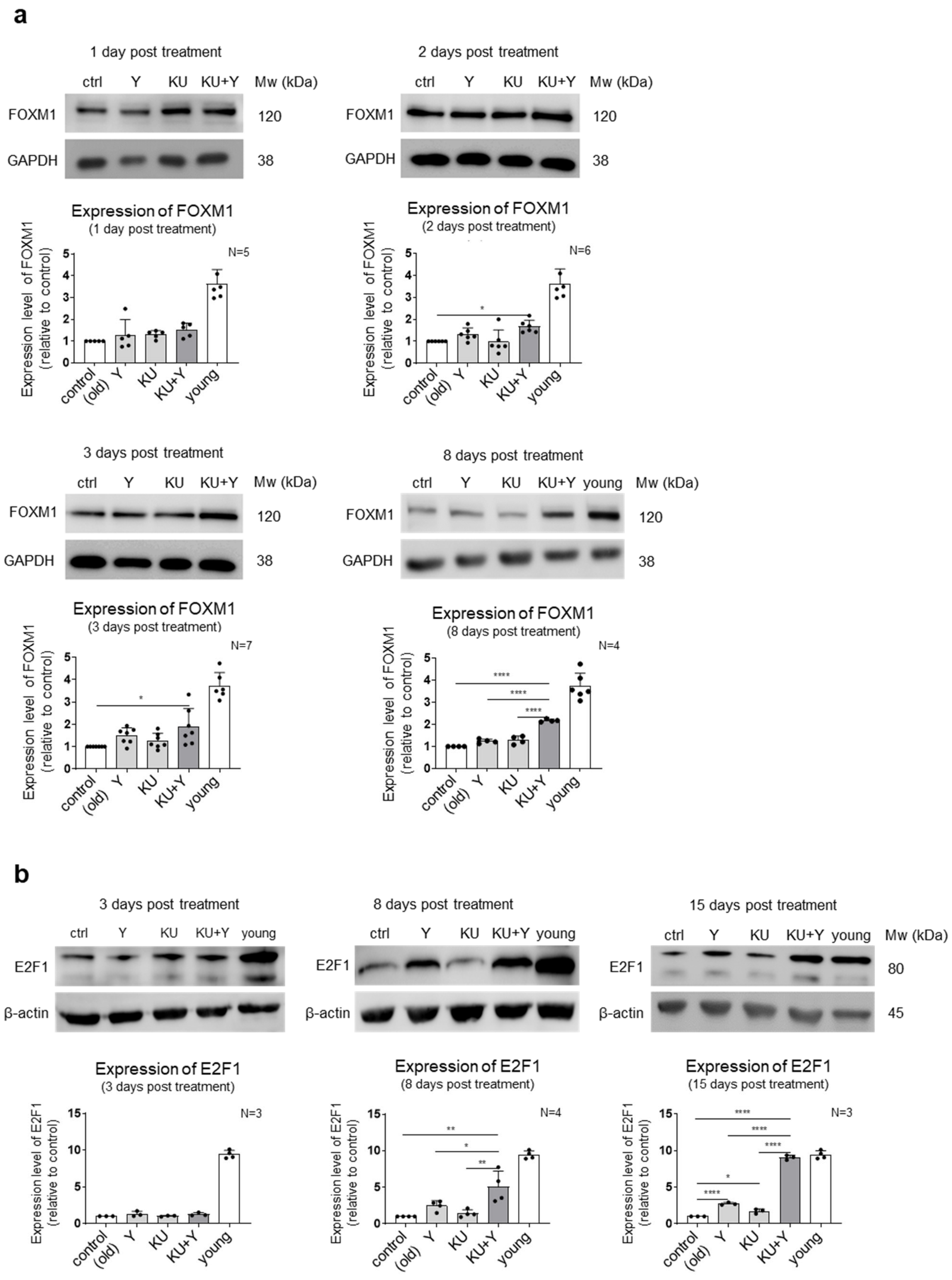
a
1 day post treatment
ctrl Y KU KU+Y Mw (kDa)
FOXM1 120
GAPDH 38
Expression of FOXM1
(1 day post treatment)
N=5
Expression level of FOXM1 (relative to control)
control (old) Y KU KU+Y young
2 days post treatment
ctrl Y KU KU+Y Mw (kDa)
FOXM1 120
GAPDH 38
Expression of FOXM1
(2 days post treatment)
N=6
3 days post treatment
ctrl Y KU KU+Y Mw (kDa)
FOXM1 120
GAPDH 38
Expression of FOXM1
(3 days post treatment)
N=7
8 days post treatment
ctrl Y KU KU+Y young Mw (kDa)
FOXM1 120
GAPDH 38
Expression of FOXM1
(8 days post treatment)
N=4
b
3 days post treatment
ctrl Y KU KU+Y young
E2F1
β-actin
8 days post treatment
15 days post treatment
ctrl Y KU KU+Y young Mw (kDa)
E2F1 80
β-actin 45
Expression of E2F1
(3 days post treatment)
N=3
Expression level of E2F1 (relative to control)
Expression of E2F1
(8 days post treatment)
N=4
Expression of E2F1
(15 days post treatment)
N=3

Supplementary Figure 10. Sequential induction of FOXM1 and E2F1 proteins by KU+Y. (**a-b**) Protein expression levels of FOXM1 (**a**) and E2F1 (**b**) measured in high passage HDFs at 1, 2, 3, 8 and 15 DPT with of KU, Y, or KU+Y. The data are shown as the mean ± s.d. values; N = 3-7 per experiment. *, $P < 0.05$; ***, $P < 1.0\times10^{-3}$; ****, $P < 1.0\times10^{-4}$ from one-way ANOVA with Tukey's post hoc correction.

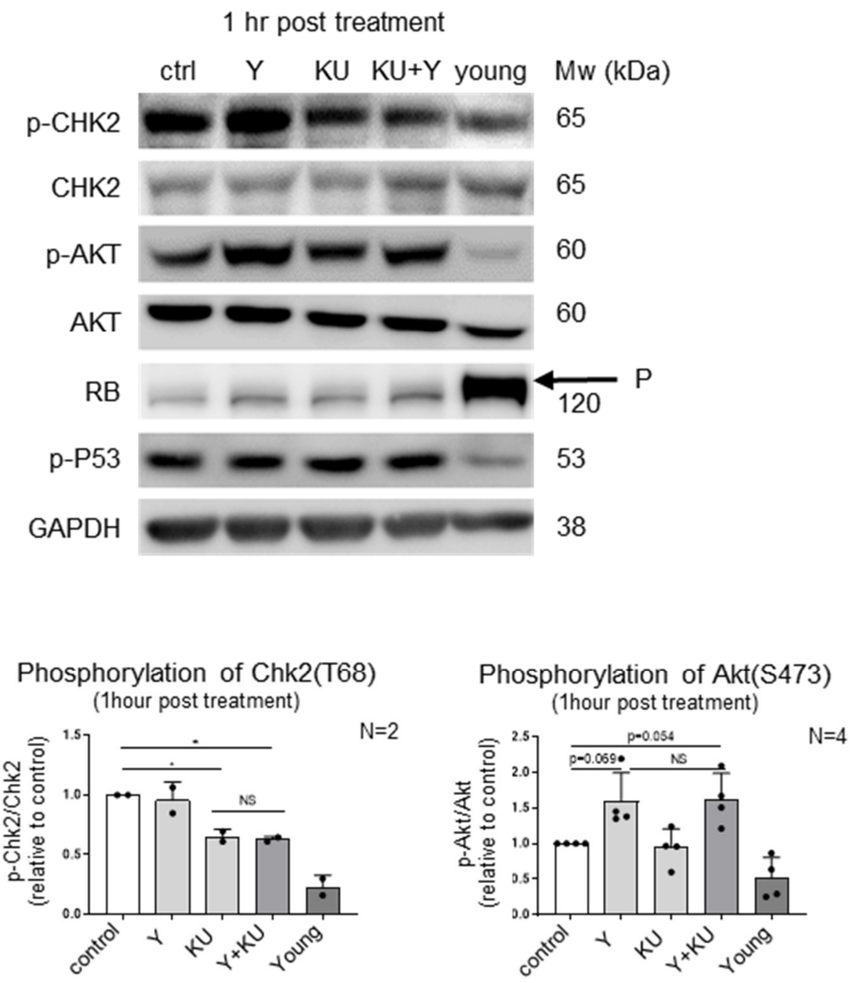

Supplementary Figure 11. KU and Y-induced phosphorylation of CHK2 and AKT. Representative immunoblot images of the indicated proteins at 1 hour after treatment with KU, Y, or KU+Y. The arrows indicate the bands for the phosphorylated form of the proteins. The phosphorylation of CHK2 (Thr68) and AKT (Ser473) were quantified. The data are shown as the mean ± s.d. values; N = 2-4 per experiment. *, $P < 0.05$ from one-way ANOVA with Tukey's post hoc correction.

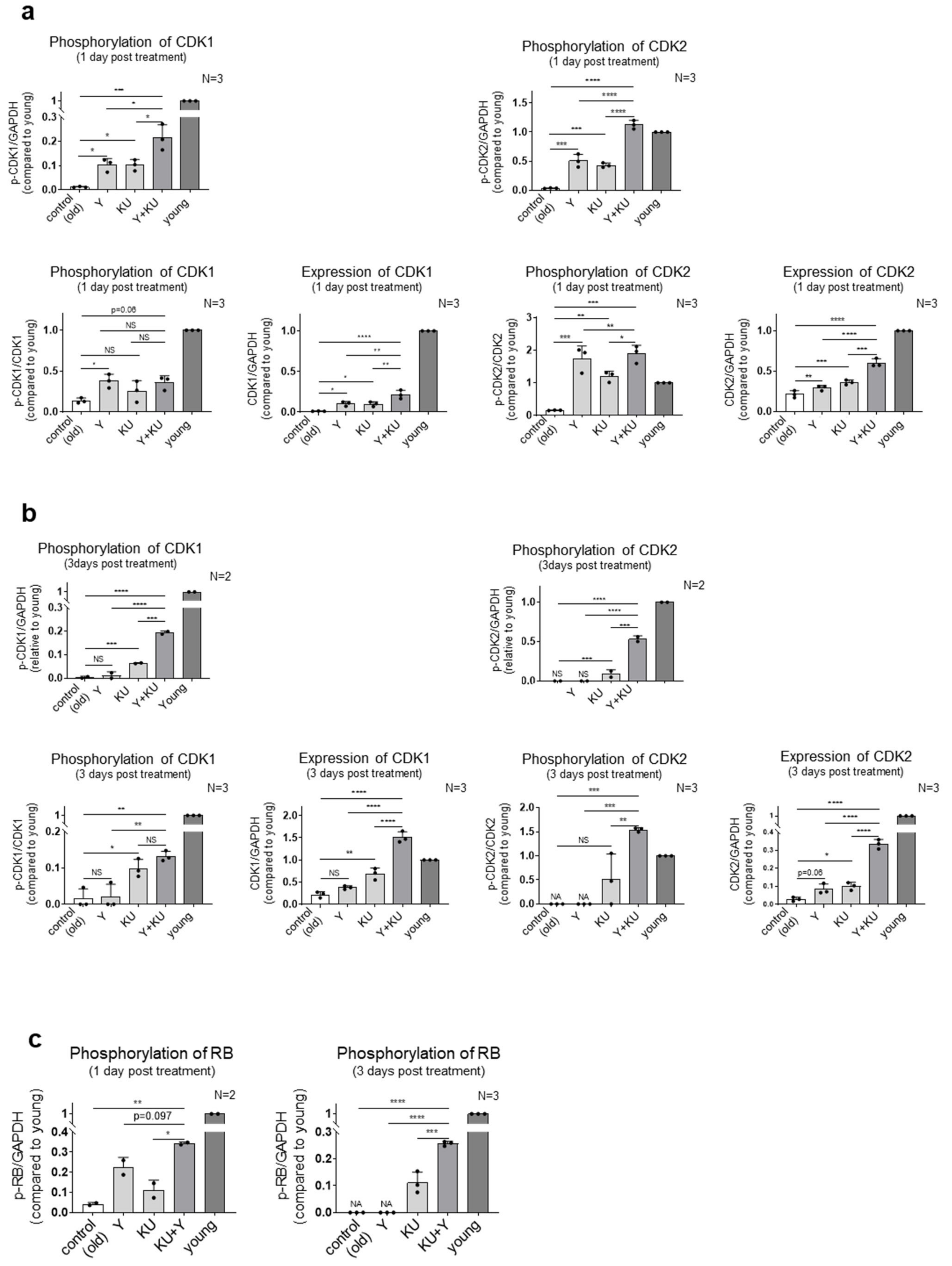
a
Phosphorylation of CDK1 (1 day post treatment)
p-CDK1/GAPDH (compared to young)
Phosphorylation of CDK2 (1 day post treatment)
p-CDK2/GAPDH (compared to young)
Phosphorylation of CDK1 (1 day post treatment)
p-CDK1/CDK1 (compared to young)
p=0.06
Expression of CDK1 (1 day post treatment)
CDK1/GAPDH (compared to young)
Phosphorylation of CDK2 (1 day post treatment)
p-CDK2/CDK2 (compared to young)
Expression of CDK2 (1 day post treatment)
CDK2/GAPDH (compared to young)
N=3
control (old)
Y
KU
Y+KU
young
b
Phosphorylation of CDK1 (3days post treatment)
p-CDK1/GAPDH (relative to young)
Phosphorylation of CDK2 (3days post treatment)
p-CDK2/GAPDH (relative to young)
N=2
Phosphorylation of CDK1 (3 days post treatment)
p-CDK1/CDK1 (compared to young)
Expression of CDK1 (3 days post treatment)
CDK1/GAPDH (compared to young)
Phosphorylation of CDK2 (3 days post treatment)
p-CDK2/CDK2 (compared to young)
Expression of CDK2 (3 days post treatment)
CDK2/GAPDH (compared to young)
p=0.06
NS
NA
c
Phosphorylation of RB (1 day post treatment)
p-RB/GAPDH (compared to young)
p=0.097
N=2
Phosphorylation of RB (3 days post treatment)
p-RB/GAPDH (compared to young)
N=3
KU+Y

Supplementary Figure 12. Quantifications of phosphorylation of cell cycle regulators. Quantification of Figure 5 showing the levels of phosphorylated PLK1 and CDC25A/B/C measured at 1 day after KU, Y, and KU+Y treatment. The levels were normalized by those of GAPDH. The data are shown as the mean ± s.d. values; N = 2-4 per experiment. *, $P < 0.05$; **, $P < 1.0\times10^{-2}$; ***, $P < 1.0\times10^{-3}$; ****, $P < 1.0\times10^{-4}$ from one-way ANOVA with Tukey's post hoc correction.

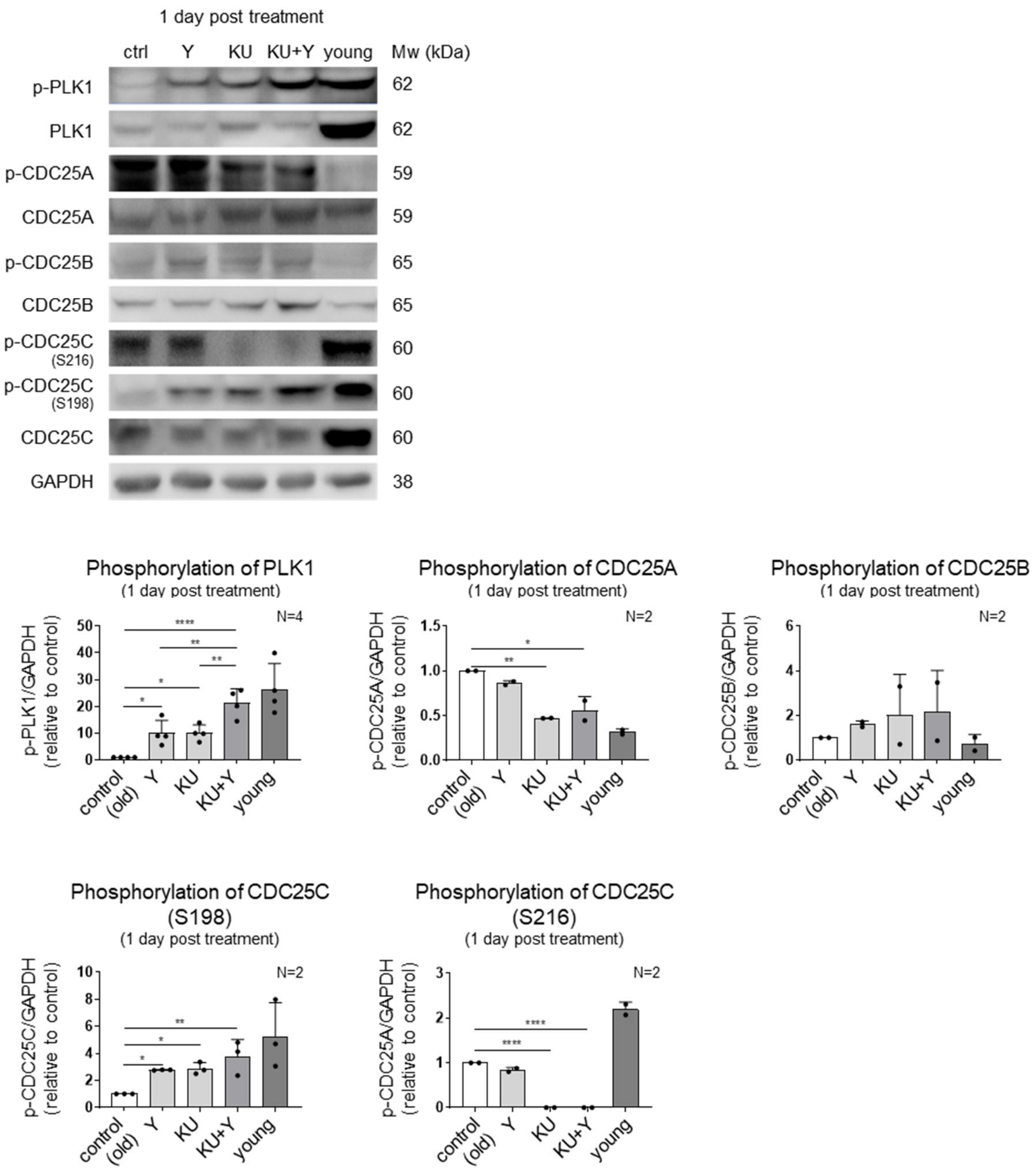

Supplementary Figure 13. Modulation of PLK1 and CDC25s by KU+Y. Representative immunoblot images of the indicated proteins at 1 day after treatment with KU, Y, or KU+Y. The phosphorylation of PLK1, CDC25A, CDC25B, and CDC25C (Ser198 and Ser216) were quantified. The data are shown as the mean ± s.d. values; N = 2 or 4 per experiment. *, $P < 0.05$; **, $P < 1.0\times10^{-2}$; ****, $P < 1.0\times10^{-4}$ from one-way ANOVA with Tukey's post hoc correction.

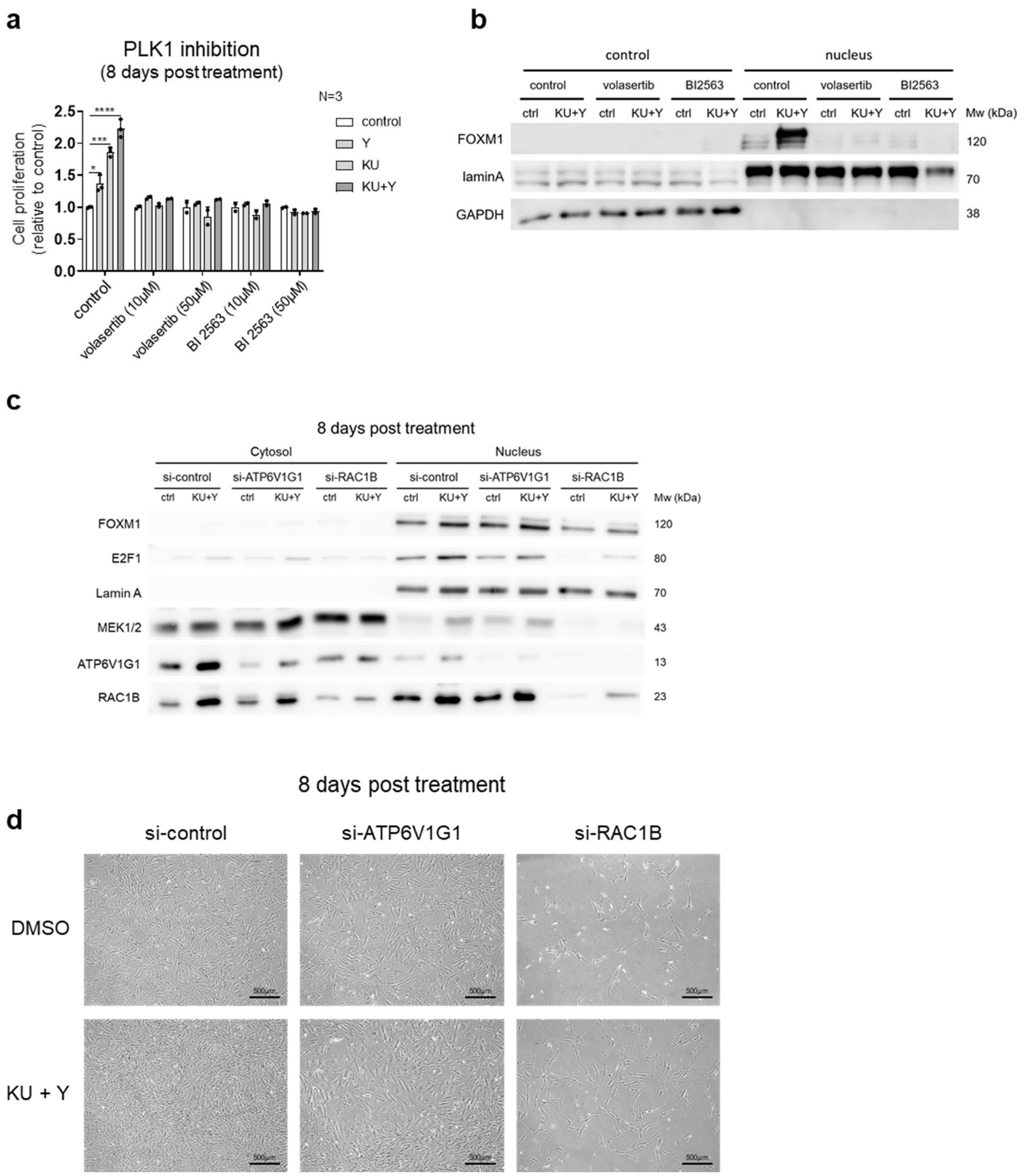

Supplementary Figure 14 PLK1-mediated synergistic effect of KU and Y on cell proliferation. (**a**) Effects of PLK1 inhibition on KU+Y-induced proliferation of high passage HDFs. The data are shown as the mean ± s.d. values; N = 3 per experiment. (**b**) Effect of PLK1 inhibition on KU+Y induced FOXM1 translocation into nucleus. (**c**) Alteration of nuclear

translocation of FOXM1 and E2F1 by knockdown of ATP6V1G1 or RAC1B. (**d**) Alteration of the effect of KU+Y on proliferation of high passage HDFs by knockdown of ATP6V1G1 or RAC1B. *, $P < 0.05$; ***, $P < 1.0\times10^{-3}$; ****, $P < 1.0\times10^{-4}$ from one-way ANOVA with Tukey's post hoc correction.

(Uncopped original western blots)

Fig. 3a

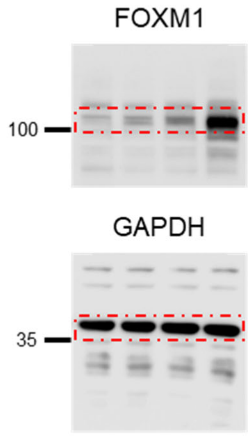

Fig. 3b

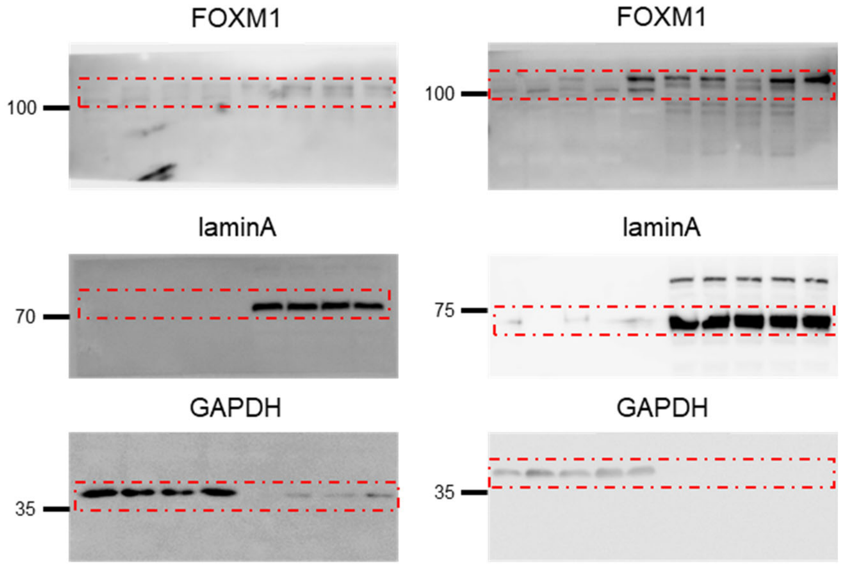

Fig. 4a

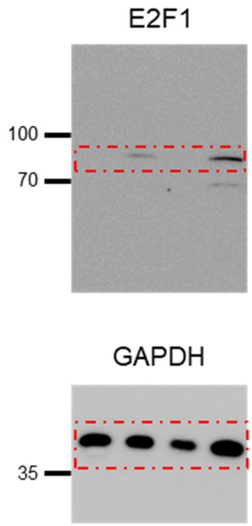

Fig. 4b

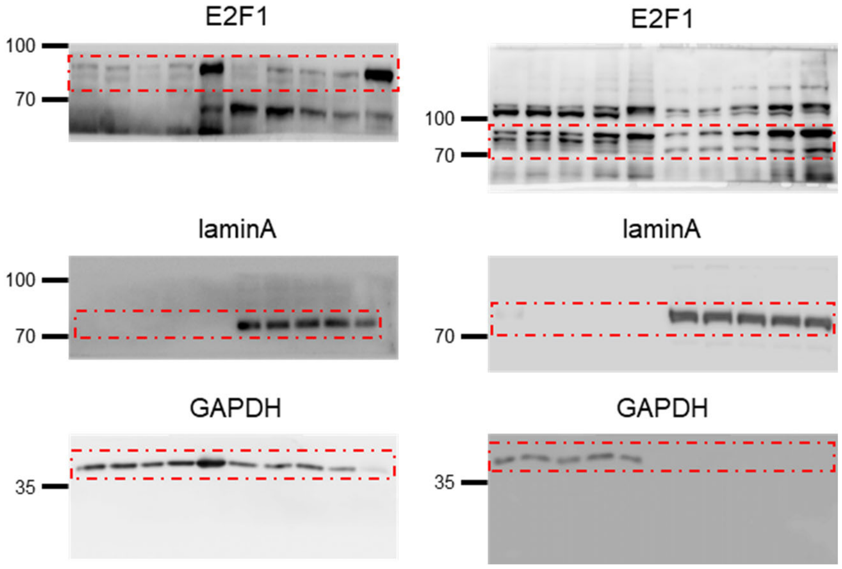

(Uncopped original western blots)

Fig. 5a

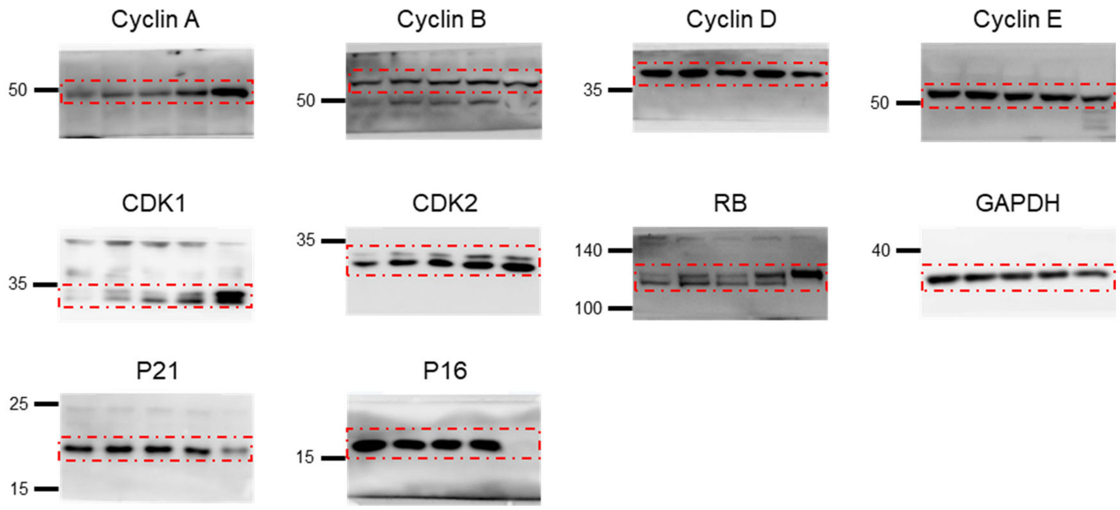

Fig. 5b

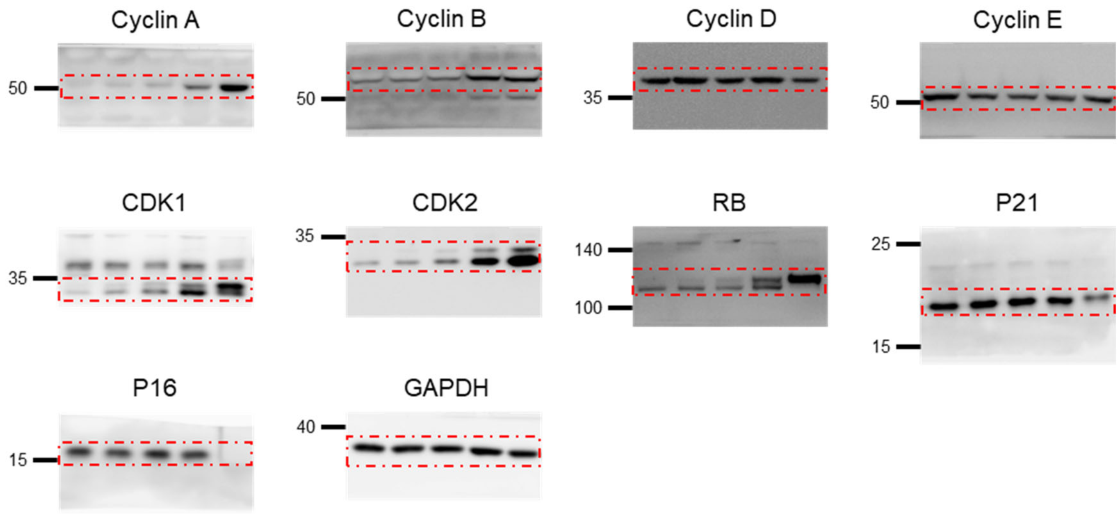

Fig. 5d

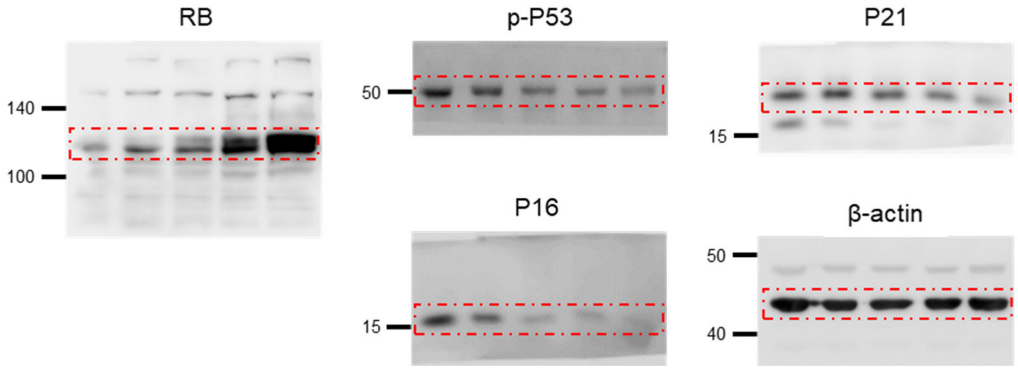

(Uncopped original western blots)

Supplementary Fig. 1

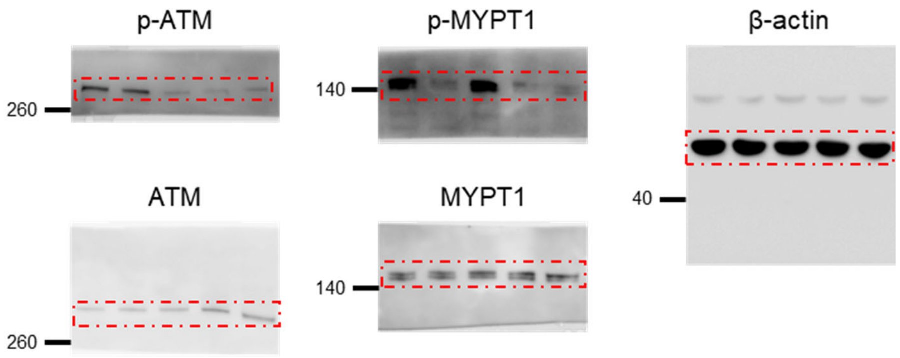

Supplementary Fig. 3j

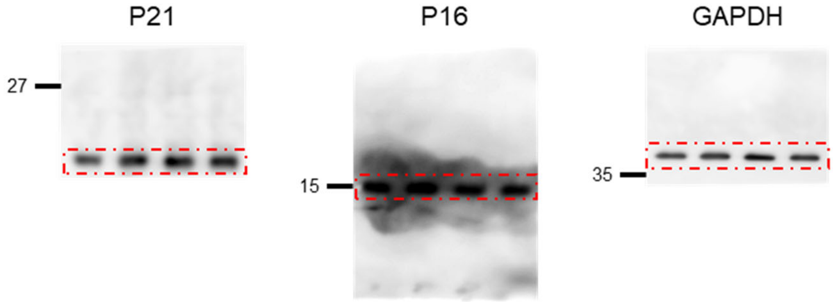

Supplementary Fig. 8

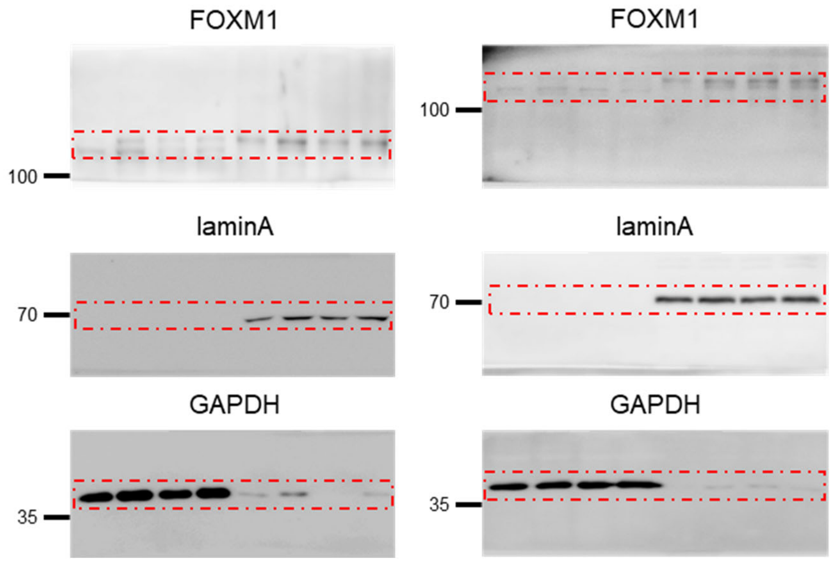

(Uncopped original western blots)

Supplementary Fig. 10

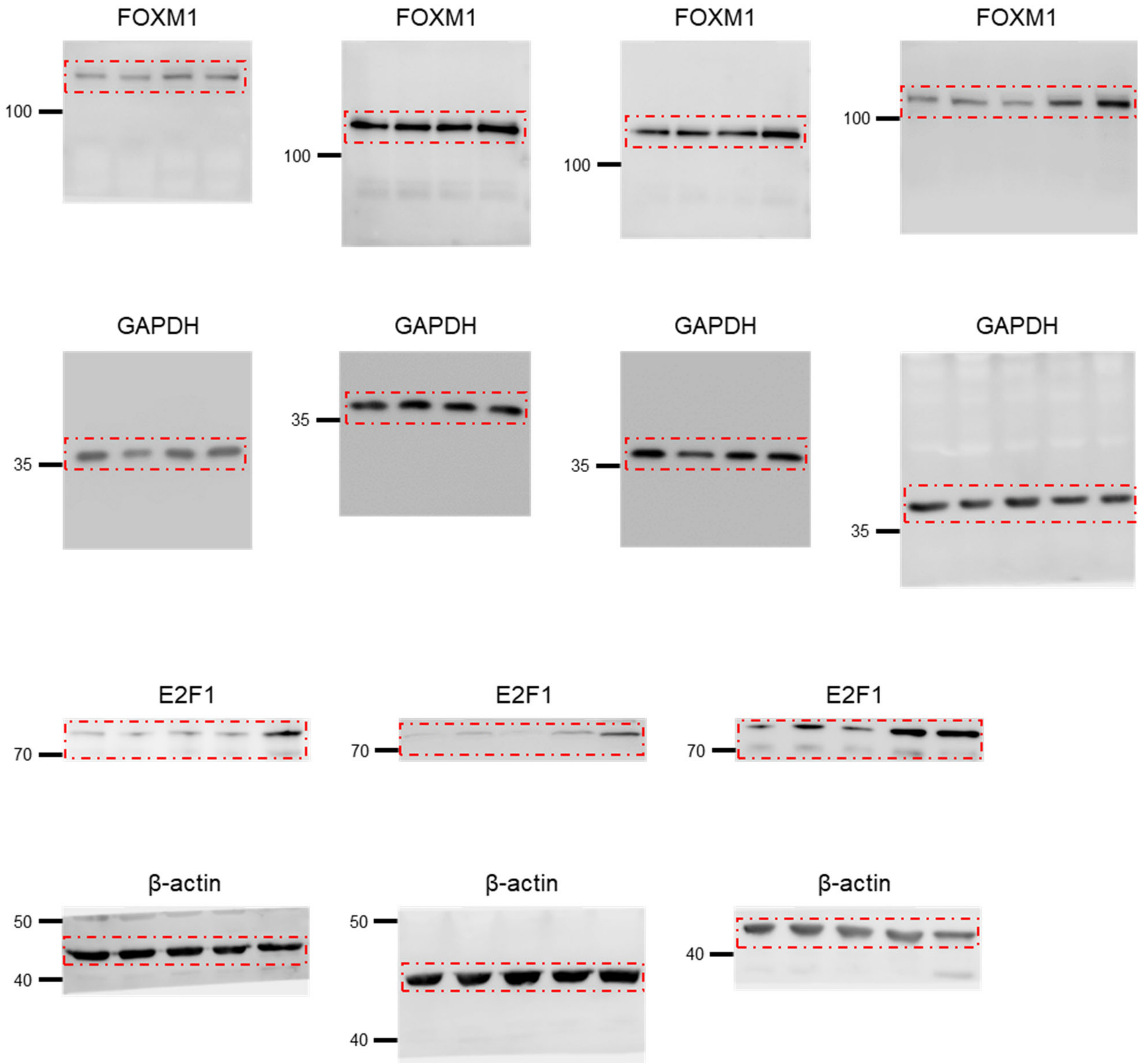

(Uncopped original western blots)

Supplementary Fig. 11

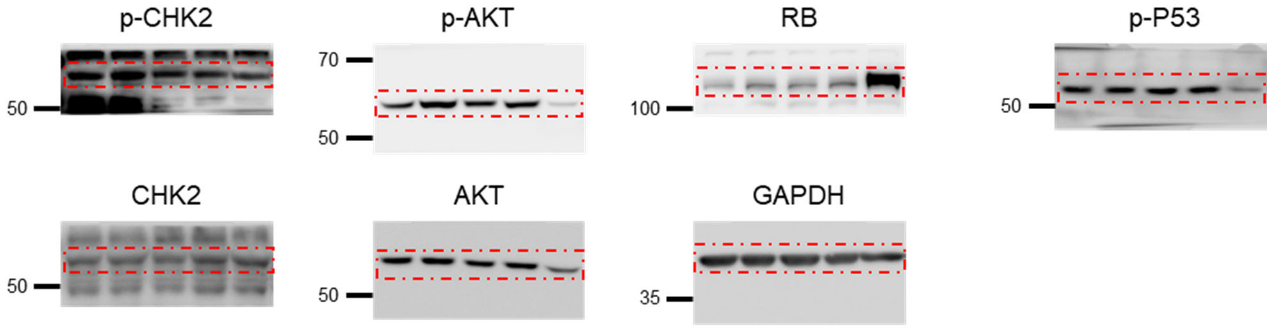

Supplementary Fig. 13

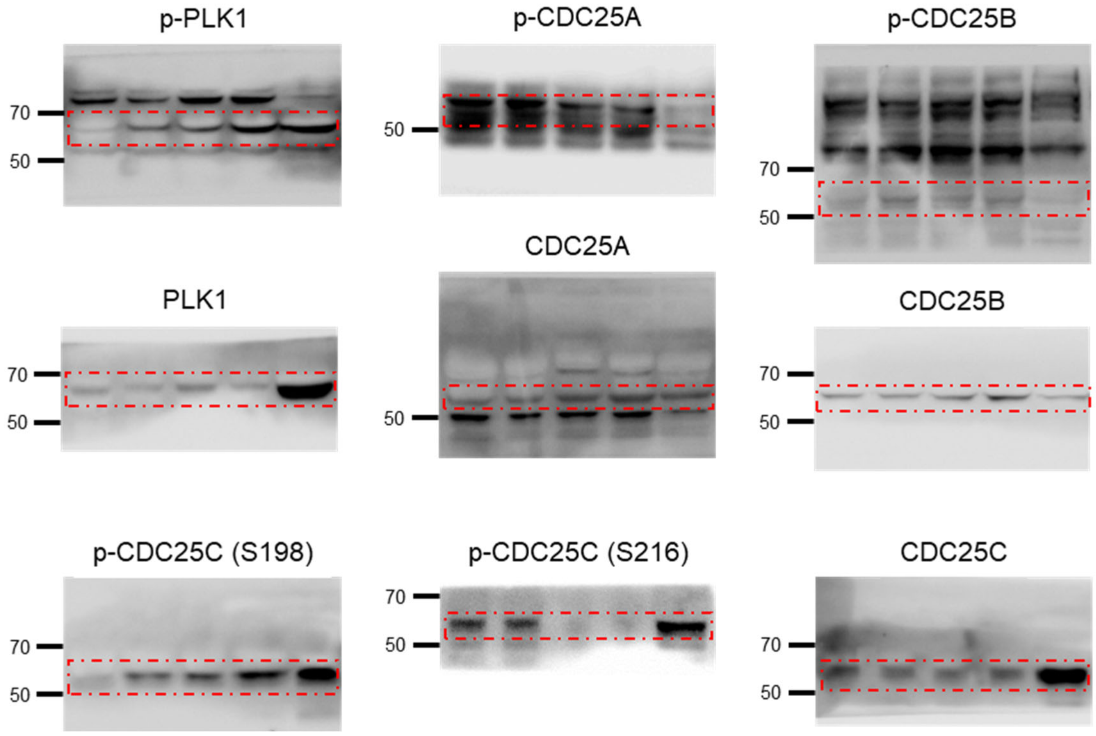

(Uncopped original western blots)

Supplementary Fig. 14b

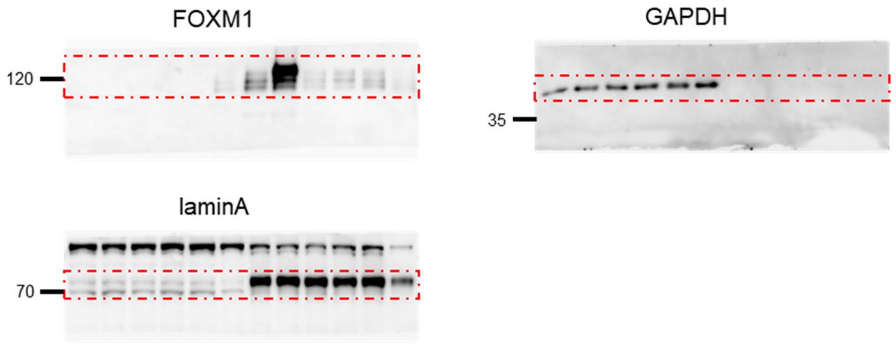

Supplementary Fig. 14c

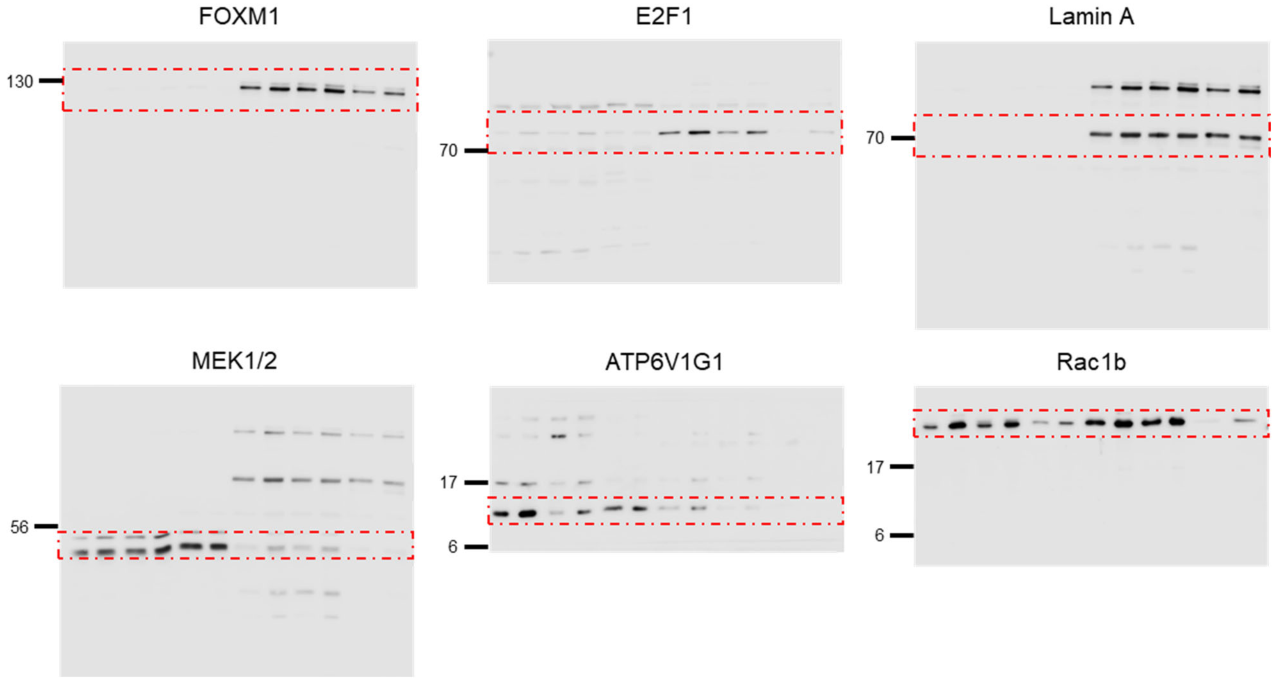

Supplement: Supplementary file 2 — Supplementary Information [file 42003_2022_3658_MOESM2_ESM.pdf]
