# Supplementary material for: Co-inhibition of ATM and ROCK synergistically improves cell proliferation in replicative senescence by activating FOXM1 and E2F1
Source: Commun Biol. 2022 Jul 14;5:702. doi: 10.1038/s42003-022-03658-5 (PMC9283421; doi:10.1038/s42003-022-03658-5)
Supplement: Supplementary file 3 — Description of Additional Supplementary Files [file 42003_2022_3658_MOESM3_ESM.pdf]

Description of Additional Supplementary Files

File name: Supplementary Data 1.

Description: Relative expression profile of SASPs measured by cytokine array. (The source data behind the graph of Fig. 1f in the paper.)

File name: Supplementary Data 2.

Description: Microarray analysis to identify SAGs, DAGs and SynAGs in each time point (3, 8 and 15 DPT). (The source data behind the graph of Fig. 2a in the paper.)

File name: Supplementary Data 3.

Description: Functional enrichment analysis result of SynAG patterns. (The source data behind the graph of Fig. 2d in the paper.)

File name: Supplementary Data 4.

Description: TF enrichment result of SynAG patterns. (The source data behind the graph of Fig. 2e in the paper.)

File name: Supplementary Data 5.

Description: List of Primer sequences used for qPT-PCR analysis. (Related to Supplementary Fig. S9a in the paper.)

File name: Supplementary Data 6.

Description: List of Primer sequences used for ChIP-qPCR analysis. (Related to the graph of Fig. 3d, 4d, and Supplementary Fig. S9b-c in the paper.)

File name: Supplementary Data 7.

Description: The numerical data that make up the all graphs in the paper.
