# Supplementary material for: Co-inhibition of ATM and ROCK synergistically improves cell proliferation in replicative senescence by activating FOXM1 and E2F1
Source: Commun Biol. 2022 Jul 14;5:702. doi: 10.1038/s42003-022-03658-5 (PMC9283421; doi:10.1038/s42003-022-03658-5)
Supplement: Supplementary file 11 — Reporting Summary [file 42003_2022_3658_MOESM11_ESM.pdf]

nature portfolio

Corresponding author(s): Young-Sam Lee (COMMSBIO-21-2211B)

Last updated by author(s): Jun 22, 2022

Reporting Summary

Nature Portfolio wishes to improve the reproducibility of the work that we publish. This form provides structure for consistency and transparency in reporting. For further information on Nature Portfolio policies, see our Editorial Policies and the Editorial Policy Checklist.

Statistics

For all statistical analyses, confirm that the following items are present in the figure legend, table legend, main text, or Methods section.

n/a	Confirmed	
☐	☒	The exact sample size (n) for each experimental group/condition, given as a discrete number and unit of measurement
☐	☒	A statement on whether measurements were taken from distinct samples or whether the same sample was measured repeatedly
☐	☒	The statistical test(s) used AND whether they are one- or two-sided<br>*Only common tests should be described solely by name; describe more complex techniques in the Methods section.*
☐	☒	A description of all covariates tested
☐	☒	A description of any assumptions or corrections, such as tests of normality and adjustment for multiple comparisons
☐	☒	A full description of the statistical parameters including central tendency (e.g. means) or other basic estimates (e.g. regression coefficient) AND variation (e.g. standard deviation) or associated estimates of uncertainty (e.g. confidence intervals)
☐	☒	For null hypothesis testing, the test statistic (e.g. F, t, r) with confidence intervals, effect sizes, degrees of freedom and P value noted<br>*Give P values as exact values whenever suitable.*
☒	☐	For Bayesian analysis, information on the choice of priors and Markov chain Monte Carlo settings
☒	☐	For hierarchical and complex designs, identification of the appropriate level for tests and full reporting of outcomes
☒	☐	Estimates of effect sizes (e.g. Cohen's d, Pearson's r), indicating how they were calculated

Our web collection on statistics for biologists contains articles on many of the points above.

Software and code

Policy information about availability of computer code

Data collection	- The robust multiarray average (RMA) method (doi:10.1093/biostatistics/4.2.249) to normalize raw data from Affymetrix array platforms obtained from publicly available dataset for cellular senescence<br>- Carl Zeiss ZEN Pro 2012 for microscope imaging<br>- i-control 2.0 for measurement of fluorescence intesity on microplate reader<br>- ImageQuant LAS 4000 for luminescence detection<br>- LightCycler 480 software 1.5.1 by Roche for qPCR data<br>- Microsoft excel 2019 for organization
Data analysis	- Custom code described in doi:10.1126/scisignal.2003266 for identifying DEGs<br>- DAVID Bioinformatics Resources 6.8 (NIAID/NIH) for performing gene ontology (GO) biological process and GO molecular function enrichment analyses and Kyoto Encyclopedia of Genes and Genomes (KEGG) pathway enrichment analysis<br>- X2Kweb (https://maayanlab.cloud/X2K/) for performing transcription factor enrichment analysis<br>- GraphPad Prism v9.0 for statistical analysis<br>- GraphPad and ggplot2 plotly and complexheatmap in R program for data visualization<br>- ImageJ (v2.0.0) for nucleus translocation<br>- ImageQuant TL (v8.2) for western blot quantification

For manuscripts utilizing custom algorithms or software that are central to the research but not yet described in published literature, software must be made available to editors and reviewers. We strongly encourage code deposition in a community repository (e.g. GitHub). See the Nature Portfolio guidelines for submitting code & software for further information.

Data

Policy information about availability of data

All manuscripts must include a data availability statement. This statement should provide the following information, where applicable:

- Accession codes, unique identifiers, or web links for publicly available datasets
- A description of any restrictions on data availability
- For clinical datasets or third party data, please ensure that the statement adheres to our policy

GSE178115 for the transcriptome analysis to examine the molecular nature of the synergistic senomorphic effects. The data will be available to public after the publication

Field-specific reporting

Please select the one below that is the best fit for your research. If you are not sure, read the appropriate sections before making your selection.

☒ Life sciences ☐ Behavioural & social sciences ☐ Ecological, evolutionary & environmental sciences

For a reference copy of the document with all sections, see nature.com/documents/nr-reporting-summary-flat.pdf

Life sciences study design

All studies must disclose on these points even when the disclosure is negative.

Sample size	At least, two biological replicates were fulfilled per treatment. Then, the results were tested for their statistical significance. The sample size of all experiments was described in the figure or figure legends.
Data exclusions	There was no any specific exclusions for the data analyses.
Replication	Every experiment was reproducible. All data presented were generated from independent biological replicates or independent experiments.
Randomization	The cell lines were obtained from public source, no pre-established selection criteria for the cell were conducted. When seeding cell for experiment, cells were distributed randomly to different well or chamber for different treatment. Images were randomly taken, then analysed. Subject population who provided samples for resource data of transcriptional changes by aging in human organ tissues or human dermal fibroblast cells was randomly selected among healthy population (Lonsdale et al., Nature Genetics. 2013; Fleischer, et al., Genome Biology. 2018).
Blinding	The investigators were not blinded to sample group allocations because changes by each treatment need to be carefully traced. Thus, blinding was not always possible during experimental setup.

Reporting for specific materials, systems and methods

We require information from authors about some types of materials, experimental systems and methods used in many studies. Here, indicate whether each material, system or method listed is relevant to your study. If you are not sure if a list item applies to your research, read the appropriate section before selecting a response.

Materials & experimental systems

n/a	Involved in the study	
☐	☒	Antibodies
☐	☒	Eukaryotic cell lines
☒	☐	Palaeontology and archaeology
☒	☐	Animals and other organisms
☒	☐	Human research participants
☒	☐	Clinical data
☒	☐	Dual use research of concern

Methods

n/a	Involved in the study	
☒	☐	ChIP-seq
☐	☒	Flow cytometry
☒	☐	MRI-based neuroimaging

Antibodies

Antibodies used	The primary antibodies used for western blot were as follows:<br>mouse anti-FOXM1 (sc-376471; 1:250 dilution; Santa Cruz Biotechnology)<br>mouse anti-E2F1 (sc-193; 1:1000 dilution; Santa Cruz Biotechnology)<br>rabbit anti-ATM (2873S; 1:1,000 dilution; Cell Signaling Technology)<br>rabbit anti-phospho-ATM (phospho-Ser1981; 4526S; 1:1,000 dilution; Cell Signaling Technology)<br>rabbit anti-MYPT1 (2634S; 1:1000 dilution; Cell Signaling Technology)

rabbit anti-phospho-MYPT1 (phosphor-Thr835; 4563S; 1:1000 dilution; Cell Signaling Technology)
rabbit anti-Chk2 (05-649; 1:500 dilution; Millipore)
rabbit anti-phospho-Chk2 (phosphor-Thr68; 2661; 1:500 dilution; Cell Signaling Technology)
rabbit anti-phospho-p53 (phosphor-Ser15; 9284; 1:500 dilution; Cell Signaling Technology)
rabbit anti-Akt (9272; 1:1,000 dilution; Cell Signaling Technology)
mouse anti-phospho-Akt (phospho-Ser473; 05-1003; 1:1,000 dilution; Millipore)
mouse anti-Retinoblastoma protein (Rb) (554136; 1:500 dilution; BD science)
rabbit anti-phosphor-PLK1 (phosphor-Thr210; 5472T; 1:1000 dilution; Cell Signaling Technology)
rabbit anti-PLK1 (4513T; 1:1000 dilution; Cell Signaling Technology)
rabbit anti-phosphor-CDC25A (phosphor-Ser124; ab156574; 1:1000 dilution; abcam)
rabbit anti-CDC25A (3652S; 1:1000 dilution; Cell Signaling Technology)
rabbit anti-phosphor-CDC25B (phosphor-Ser353; orb571847; 1:1000 dilution; biorbyt)
rabbit anti-CDC25B (9525; 1:1000 dilution; Cell Signaling Technology)
rabbit anti-phosphor-CDC25C (phosphor-Ser216; 4901T; 1:1000 dilution; Cell Signaling Technology)
rabbit anti-phosphor-CDC25C (phosphor-Ser198; 9529T; 1:1000 dilution; Cell Signaling Technology)
rabbit anti-CDC25C (4688T; 1:1000 dilution; Cell Signaling Technology)
rabbit anti-cyclin A (sc-751; 1:200 dilution; Santa Cruz Biotechnology)
mouse anti-cyclin B (sc-245; 1:250 dilution; Santa Cruz Biotechnology)
mouse anti-cyclin D (sc-246; 1:200 dilution; Santa Cruz Biotechnology)
mouse anti-cyclin E (sc-247; 1:250 dilution; Santa Cruz Biotechnology)
rabbit anti-phophor-CDK1 (phospho-Thr161; 9114S; 1:1,000 dilution; Cell Signaling Technology)
rabbit anti-phophor-CDK2 (phospho-Thr160; 2561S; 1:1,000 dilution; Cell Signaling Technology)
rabbit anti-p21 (2947S; 1:1,000 dilution; Cell Signaling Technology)
rabbit anti-p16 (ab108349; 1:1,000 dilution; Abcam)
rabbit anti-laminA (ab26300; 1:1000 dilution; Abcam)
mouse anti-GAPDH (G041; 1:1,000 dilution; ABM)

Validation

All antibodies are from commercially available and have been validated from the manufacturer with supporting publications linked on manufacturer's websites as follows:
mouse anti-FOXM1 (sc-376471; 1:250 dilution; Santa Cruz Biotechnology), (https://www.scbt.com/p/foxm1-antibody-g-5)
mouse anti-E2F1 (sc-193; 1:1000 dilution; Santa Cruz Biotechnology), (https://www.scbt.com/p/e2f-1-antibody-c-20)
rabbit anti-ATM (2873S; 1:1,000 dilution; Cell Signaling Technology), (https://www.cellsignal.com/products/primary-antibodies/atm-d2e2-rabbit-mab/2873)
rabbit anti-phospho-ATM (phospho-Ser1981; 4526S; 1:1,000 dilution; Cell Signaling Technology), (https://www.cellsignal.com/products/primary-antibodies/phospho-atm-ser1981-10h11-e12-mouse-mab/4526)
rabbit anti-MYPT1 (2634S; 1:1000 dilution; Cell Signaling Technology), (https://www.cellsignal.com/products/primary-antibodies/mypt1-antibody/2634)
rabbit anti-phospho-MYPT1 (phosphor-Thr835; 4563S; 1:1000 dilution; Cell Signaling Technology), (https://www.cellsignal.com/products/primary-antibodies/phospho-mypt1-thr853-antibody/4563)
rabbit anti-Chk2 (05-649; 1:500 dilution; Millipore), (https://www.merckmillipore.com/product/Anti-Chk2-Antibody-clone-7,MM_NF-05-649)
rabbit anti-phospho-Chk2 (phosphor-Thr68; 2661; 1:500 dilution; Cell Signaling Technology), (https://www.cellsignal.com/products/primary-antibodies/phospho-chk2-thr68-antibody/2661)
rabbit anti-phospho-p53 (phosphor-Ser15; 9284; 1:500 dilution; Cell Signaling Technology), (https://www.cellsignal.com/products/primary-antibodies/phospho-p53-ser15-antibody/9284)
rabbit anti-Akt (9272; 1:1,000 dilution; Cell Signaling Technology), (https://www.cellsignal.com/products/primary-antibodies/akt-antibody/9272)
mouse anti-phospho-Akt (phospho-Ser473; 05-1003; 1:1,000 dilution; Millipore), (https://www.merckmillipore.com/product/Anti-phospho-Akt-Ser473-Antibody-clone-6F5,MM_NF-05-1003)
mouse anti-Retinoblastoma protein (Rb) (554136; 1:500 dilution; BD science), (https://www.bdbiosciences.com/en-us/products/reagents/flow-cytometry-reagents/research-reagents/single-color-antibodies-ruo/purified-mouse-anti-human-retinoblastoma-protein.554136)
rabbit anti-phosphor-PLK1 (phosphor-Thr210; 5472T; 1:1000 dilution; Cell Signaling Technology), (https://www.cellsignal.com/products/primary-antibodies/phospho-plk1-thr210-antibody/5472)
rabbit anti-PLK1 (4513T; 1:1000 dilution; Cell Signaling Technology), (https://www.cellsignal.com/products/primary-antibodies/plk1-208g4-rabbit-mab/4513)
rabbit anti-phosphor-CDC25A (phosphor-Ser124; ab156574; 1:1000 dilution; abcam), (https://www.abcam.com/cdc25a-phospho-s124-antibody-epr8888-ab156574.html)
rabbit anti-CDC25A (3652S; 1:1000 dilution; Cell Signaling Technology), (https://www.cellsignal.com/products/primary-antibodies/cdc25a-antibody/3652)
rabbit anti-phosphor-CDC25B (phosphor-Ser353; orb571847; 1:1000 dilution; biorbyt), (https://www.biorbyt.com/cdc25b-phospho-ser353-antibody-orb571847.html)
(rabbit anti-CDC25B (9525; 1:1000 dilution; Cell Signaling Technology), (https://www.cellsignal.com/products/primary-antibodies/cdc25b-antibody/9525)
rabbit anti-phosphor-CDC25C (phosphor-Ser216; 4901T; 1:1000 dilution; Cell Signaling Technology), (https://www.cellsignal.com/products/primary-antibodies/phospho-cdc25c-ser216-63f9-rabbit-mab/4901)
rabbit anti-phosphor-CDC25C (phosphor-Ser198; 9529T; 1:1000 dilution; Cell Signaling Technology), (https://www.cellsignal.com/products/primary-antibodies/phospho-cdc25c-ser198-antibody/9529)
rabbit anti-CDC25C (4688T; 1:1000 dilution; Cell Signaling Technology), (https://www.cellsignal.com/products/primary-antibodies/cdc25c-5h9-rabbit-mab/4688)
rabbit anti-cyclin A (sc-751; 1:200 dilution; Santa Cruz Biotechnology), (https://www.scbt.com/p/cyclin-a-antibody-h-432)
mouse anti-cyclin B (sc-245; 1:250 dilution; Santa Cruz Biotechnology), (https://www.scbt.com/p/cyclin-b1-antibody-gns1)
mouse anti-cyclin D (sc-246; 1:200 dilution; Santa Cruz Biotechnology), (https://www.scbt.com/ko/p/cyclin-d1-antibody-hd11)
mouse anti-cyclin E (sc-247; 1:250 dilution; Santa Cruz Biotechnology), (https://www.scbt.com/ko/p/cyclin-e-antibody-he12)
rabbit anti-phophor-CDK1 (phospho-Thr161; 9114S; 1:1,000 dilution; Cell Signaling Technology), (https://www.cellsignal.com/products/primary-antibodies/phospho-cdc2-thr161-antibody/9114)

rabbit anti-phophor-CDK2 (phospho-Thr160; 2561S; 1:1,000 dilution; Cell Signaling Technology), (https://www.cellsignal.com/products/primary-antibodies/phospho-cdk2-thr160-antibody/2561)
rabbit anti-p21 (2947S; 1:1,000 dilution; Cell Signaling Technology), (https://www.cellsignal.com/products/primary-antibodies/p21-waf1-cip1-12d1-rabbit-mab/2947)
rabbit anti-p16 (ab108349; 1:1,000 dilution; Abcam), (https://www.abcam.com/cdkn2ap16ink4a-antibody-epr1473-c-terminal-ab108349.html)
rabbit anti-laminA (ab26300; 1:1000 dilution; Abcam), (https://www.abcam.com/lamin-a-antibody-ab26300.html)
mouse anti-GAPDH (G041; 1:100,000 dilution; ABM), (https://www.abmgood.com/g041-1mgname.html)
mouse anti-β-Actin (sc-47778; 10,000 dilution; Santa Cruz), (https://www.scbt.com/p/beta-actin-antibody-c4)

Eukaryotic cell lines

Policy information about cell lines

Cell line source(s)	Human Primary Dermal Fibroblast purchased from ATCC (cat. number PCS-201-010)
Authentication	The cell lines were obtained from public source, so that none of the cell lines used were authenticated.
Mycoplasma contamination	Cells were tested for mycoplasma contamination with a MycoAlert Mycoplasma Detection Kit (LT07-318; Lonza).
Commonly misidentified lines (See ICLAC register)	(none)

Flow Cytometry

Plots

Confirm that:

☒ The axis labels state the marker and fluorochrome used (e.g. CD4-FITC).

☒ The axis scales are clearly visible. Include numbers along axes only for bottom left plot of group (a 'group' is an analysis of identical markers).

☒ All plots are contour plots with outliers or pseudocolor plots.

☒ A numerical value for number of cells or percentage (with statistics) is provided.

Methodology

Sample preparation	After washing twice with PBS, cells were trypsinized, harvested in 500 µl of PBS, and injected into an LSR Fortessa (Beckton Dickson) flow cytometer.
Instrument	LSRFortessa (manufactured by BD)
Software	BD FACSDiva software (version 7.0)
Cell population abundance	The number of cell population was around 10,000 cells for MitoSOX and TMRM; 5,000 cells for MitoTimer; 30,000 cells for PI staining.
Gating strategy	There is no special the preliminary FSC/SSC gates of the starting cell population.<br>- Autofluorescence from lipofuscin was acquired at an excitation wavelength of 488 nm with a 530/30 nm bandpass filter (FITC channel).<br>- Signals from Mitotracker (30 micromolar) stained cells were acquired at an excitation wavelength of 488 nm with a 530/30 nm bandpass filter (FITC channel).<br>- Signals from MitoSOX (5 micromolar) stained cells were acquired at an excitation wavelength of 561 nm with a 615/24 nm bandpass filter (PE-Texas Red channel).<br>- Signals from TMRM (1X, by Invitrogen; #I34361) stained cells were acquired at an excitation wavelength of 561 nm with a 583/22 nm bandpass filter (PE channel).<br>- Signals from the MitoTimer reporter transduced cells were acquired simultaneously at the excitation wavelength of 488 nm with a 530/30 nm bandpass filter (FITC channel) and at the excitation wavelength of 561 nm with a 583/22 nm bandpass. filter, PE channel).<br>- Signals from the Propidium Iodide (PI) stained cells were acquired at the excitation wavelength of 561 nm with a 615/24 nm bandpass filter (PE-Texas Red channel).

☒ Tick this box to confirm that a figure exemplifying the gating strategy is provided in the Supplementary Information.
